# How to improve technical and tactical actions of dominant and non-dominant players in children’s football?

**DOI:** 10.1371/journal.pone.0254900

**Published:** 2021-07-22

**Authors:** Mirjam Hintermann, Dennis-Peter Born, Jörg Fuchslocher, Raphael Kern, Michael Romann

**Affiliations:** 1 Department for Elite Sport, Swiss Federal Institute of Sport Magglingen, Magglingen, Switzerland; 2 Swiss Football Association, Muri bei Bern, Switzerland; 3 Swiss Swimming Federation, Bern, Switzerland; University of Innsbruck, AUSTRIA

## Abstract

As young football players develop important technical and tactical skills during competitive matches, this study investigated quantity and quality of technical and tactical actions in real game conditions in a 4v4 compared to the traditional 7v7 match format. In total, three matches of each format were played by 103 young football players (10.3±0.6 years) and video monitored for subsequent manual tagging of technical and tactical events. Based on the number of technical and tactical actions in the 7v7 matches, players were classified as dominant or non-dominant and changes in these subgroups were assessed during the 4v4 match format. The 4v4 match format significantly (*P*<0.001) increased total number of actions per player per minute compared to the 7v7 matches (5.59±1.44 and 2.78±0.73, respectively) and the number of successful (2.88±0.92 and 1.15±0.49, respectively) and unsuccessful (1.05±0.42 and 0.67±0.23, respectively) actions. Both dominant and non-dominant players increased their number of actions during the 4v4 compared to 7v7 match format. Despite a missing significant interaction effect, there was a larger percentage increase in number of actions for the non-dominant players (143%) compared to dominant players (72%) in 4v4. The 4v4 match format shows twice as many technical and tactical actions in real game conditions and, therefore, may improve players’ skill development.

## Introduction

During a football match, players must continuously decide between a wide variety of complex technical and tactical actions within fractions of a second [1]. For successful decision-making, cognitive skills such as quick understanding of game situations, anticipation of the opponent’s actions, and intuitive decision-making are needed. These skills require the combination of automated thinking and coordinative movement patterns, which is preferably obtained in real game situations [2, 3]. Therefore, engaging in a high number of free play and football specific deliberate play activities is important for athlete development [4–7]. As such, match formats in children’s football (5–10 years) should maximize game involvement and on-ball actions of all players [8], and allow flexible playing positions.

Small-sided games (SSG) have been introduced as an additional tool to traditional match formats with the aim to improve player selection and player development. SSG are played on reduced pitch sizes and with reduced number of players, they are well-known, and scientifically proven to increase players’ game participation [9]. The SSG are often used to increase the physiological load on each player and to simulate key situations from previous or upcoming games [9, 10]. Recent studies showed that SSG may improve technical and tactical skills during talent development of young football players, mainly evaluated during training. For instance, reduced pitch size and fewer players have been shown to result in increased game involvement and greater frequency of individual actions, i.e., passes, dribblings, and shots [11–15]. Additionally, the number of attempts to score were increased by changing the targets, such as scoring points by crossing an end line or removing the goalkeeper, while decreasing the size of the goal but adding an additional goal at each end of the pitch [16]. Furthermore, additional goals also increased ball movement in the lateral pitch areas and decreased central field play [17].

While SSG may be beneficial for some qualitative and quantitative technical and tactical actions, larger pitch sizes increase position specific demands, movements across a wide range of width and depth of the pitch, as well as increased number of long passes [15, 18]. The larger number of players involved in match format on larger pitches increased team tactical actions, as well as defensive coverage between off-ball defenders in response to movements of attackers [18]. However, playing on a larger pitch decreased number of goals, dribblings, and interceptions [19, 20].

In addition to pitch size and number of players, various factors affected game involvement in children’s football. For instance, players born later in the year showed less technical and tactical actions compared to their peers born earlier in the same year [21]. Additionally, late maturing and biologically younger players may also be less involved in the technical and tactical actions [22]. As talent development aims for a holistic development in a large number of talents [23], match formats should maximize technical and tactical actions, as well as game involvement of all players. In line with the literature, the Swiss Football Association (SFA) has specified in the children’s football concept that all players should be given the opportunity to have a high level of match participation [8]. There is anecdotal evidence among SFA coaches that this is not the case in 7v7, as most teams have dominant players who shape the game on the one hand and non-dominant players who have little game action on the other. Due to the practical relevance and the fact that the actions of the individual player increase when the playing field is reduced, the inter-individual variation and the difference between dominant and non-dominant players should be analysed. Additionally, match formats should create task-specific development opportunities and facilitate quick decision-making skills. As a larger total number of actions are hypothesized for matches on smaller pitch sizes, differences in game involvement between early and late maturing players may be reduced [24].

While both, small-sided games and competitive matches on large pitches, may provide important learning opportunities in children’s football, recent studies mainly evaluated small-sided games during training [9, 15]. However, expertise from competitive matches provide the best opportunities for task-specific development of technical and tactical skills, including cognitive abilities and quick decision-making skills [25]. Therefore, the aim of the present study was to investigate 1) the quantity and 2) the quality of the technical and tactical actions, as well as 3) game involvement of dominant and non-dominant players during competitive matches of a 4v4 small sided game compared to the traditional 7v7 match format in Swiss children’s football. The hypotheses were that the 4v4 match format would 1) increase the number of actions per player, 2) improve the quality of actions, i.e. successful versus unsuccessful actions, and 3) would enable non-dominant players to complete more actions compared to the traditional 7v7 match format.

## Methods

### Participants

For the present study, Swiss football U11 players (n = 132) were recruited from 16 teams to play an organized tournament including both the traditional 7v7 and the 4v4 match format. All participants participated in regular football practice of two training sessions and one competitive match per week for 3.1 ± 0.6 years. All participants played in the second highest division for this age group. Players who did not reach a minimum of 5.22 playing minutes (mean minus one standard deviation) in both match formats were excluded (n = 29). Therefore, 103 players (age: 10.3 ± 0.6 years), including 12 girls (age: 11.3 ± 0.4) and 91 boys (age: 10.2 ± 0.6), were analyzed. Differences in age between sexes is based on the national football association SFA regulations and common practice, where girls are allowed to be one year older when competing with the boys in this age group. Written informed consent of participation was signed by all players and their legal guardians. The study was approved by the institutional review board of the Swiss Federal Institute of Sport Magglingen (021_LSP_10_07_17) and was in accordance with the Declaration of Helsinki.

### Study design

Data were collected during four single-day football tournaments, which took place on natural outdoor grass pitches, in dry conditions between 9 and 11 am. The 16 participating teams were randomly allocated to one of these four tournaments. Each tournament lasted 2.5 h and included three 4v4 and three 7v7 matches. The 4v4 matches were played on a 30 x 20 meter pitch (75 m^2^ per player), as 3 x 10 minutes matches with two minutes breaks between each match. The 7v7 were played on a 50 x 30 meter pitch (107 m^2^ per player), as 3 x 20 minutes matches with five minutes breaks between each match. Pitch sizes of 7v7 were like traditional match formats according to SFA rules. Pitch dimensions of 4v4 matches were derived from applied practice in Switzerland and according to literature [9, 26]. Between the two match formats players had a longer rest period of 15 minutes to reorganize the playing fields from four 4v4 pitches into two 7v7 pitches. All matches were video recorded for subsequent analysis of technical and tactical actions. Data were analyzed by three expert analysts of the national football association with high coaching and scouting experience in children’s football (17.7 ± 2.5 years). Video footages were stored and accessed using the cloud solution of Dartfish TV and Dartfish video tagging tool (Dartfish Note, Dartfish, Fribourg, Switzerland).

### Match formats

The 4v4 match format was developed by an expert panel including coaches and technical staff of the national football association, based on previous research findings [9, 27]. For the 4v4 matches, coaches were asked to divide their players into two equally strong subteams. The 4v4 match format was played with four small goals (0.8 x 1.2 m) that were positioned in pairs of two at each end (short side) of the pitch. No goalkeeper was involved in the 4v4 match format. To ensure a fast and fluid match, the game was re-initiated using the spare balls by a kick-in from the sideline or a corner kick from between the two small goals when the ball went out. For practical reasons, the player who was playing the ball off the pitch, retrieved the ball immediately. The remaining players continued playing with the spare balls available in marked zones of each side. The 7v7 control matches, were played according to traditional rules for this age group. There were 6 players and one goalkeeper per team, and the goal dimensions were 2 x 5 meters. When the ball went out during the 7v7 match, the goalkeepers replaced it with one of the spare balls placed in their goal to assure a quick and fluid game. Both match formats were played without the offside rule or a referee.

### Data collection

All 7v7 matches were video monitored from both sidelines using two digital cameras (HDR-CX700VE, Sony, Minato, Tokyo, Japan). The digital cameras were positioned on a tripod 4 m from the long side of the pitch and 22 m from the short side. Matches were video-captured ball-oriented (focused on the ball) by trained expert analysts of the national football association. Additionally, a dome camera monitored the entire pitch from 8 m behind the short side, and was positioned on a six meter high tripod.

### Pilot study

As the 4v4 matches were scheduled prior to the 7v7 matches in every tournament, a pilot study was conducted to rule out a possible fatigue effect. For the pilot study, 165 young football players (age: 10.2 ± 0.7 years), who were not involved in the actual intervention study, played the traditional 7v7 with no prior matches. Comparison of intervention and pilot study showed no differences in the total number of actions (*P* = 0.33), ball controls (*P* = 0.93), passes (*P* = 0.20), dribblings (*P* = 0.37), runs with ball (*P* = 0.22), duels (*P* = 0.14), interceptions (*P* = 0.11) and pressing (*P* = 0.28) with or without previous games. However, there were a greater number of shots (*P* = 0.03) in the intervention condition. Additionally, for the intervention study, a one-way ANOVA was calculated and revealed no significant differences between the total number of technical and tactical actions between the first, second and third match in 4v4 (*P* = 0.87, *F*_2,343_ = 0.14) and 7v7 (*P* = 0.82, *F*_2,289_ = 0.20).

### Data analysis

Before the video analysis, all three expert raters were informed of the study design and the specifics of the parameters. They analyze 12 matches using the tagging-panel for familiarization. During the familiarization phase, results of the analyses were discussed by the expert panel to insure consistent assessment of technical and tactical actions and their quality among the three raters. To assess interrater reliability, all technical and tactical actions of 28 randomly selected players were analyzed by two of the raters. For the final analyses, the mean values were taken. Intraclass correlation coefficient for total number of actions showed good (0.89) reliability [28] (values of single items: ball controls = 0.65, passes = 0.99, dribblings = 0.89, runs with ball = 0.67, shots = 0.99, duels = 0.70, interceptions = 0.78 and pressing = 0.71). To assess the test-retest reliability, seven games were randomly selected and analyzed in duplicate by each of the raters. Pearson’s correlation coefficient (mean of all parameters analyzed by each rater) indicated excellent inter-rater reliability (rater 1 (r = 0.82), rater 2 (r = 0.75) and rater 3 (r = 0.94)).

For the present study, players were analyzed individually for a paired comparison between the 4v4 and 7v7 match format. For the offensive phases, parameters of interest were defined and passes, ball controls, dribblings, runs with ball and shots analyzed. During defensive phases duels, interceptions and pressing was analyzed. In addition to the quantitative analysis, actions were assessed on their quality of execution, i.e., quality of ball controls (successfully ball control after one touch), passes (successfully executed pass with right direction of the ball), dribblings (successfully dribbling while under pressure from an attacking opponent), shots (successful shot leading to a goal) and duels (successful duel followed by ball possession). The total number of action was assessed adding up all offensive and defensive technical and tactical actions per player. Playing time per player was manually recorded during tagging. All analyzed technical and tactical actions were expressed per player per minute for a valid comparison between the two match formats.

To investigate game involvement, all players were ranked based on the mean of total number of actions in the 7v7 match format (S1 Fig). Three groups were defined from the total number of actions, whereby the middle third (n = 35) was not considered for this evaluation. The upper third of players with the largest number of actions were labeled as dominant (n = 34), while the lower third of players with the smallest number of actions was labelled as non-dominant (n = 34). Game involvement of dominant and non-dominant players were compared across the 4v4 and 7v7 match formats.

### Statistical analysis

All data are presented as mean ± SD, with an alpha-level of < 0.05 indicating statistical significance. Normality was assessed through visual inspection. The predicted and standardized residuals showing a random distribution around zero in the scatter plot, a diagonal straight line in the normal probability plot and a Gaussian distribution in the histogram (S1 Fig) [29]. The match formats, 4v4 and 7v7, were compared using paired *t*-tests. To assess the practical relevance of differences, effect sizes were calculated according to Cohen using the means and pooled standard deviations [30]. Cohen’s *d* effect sizes of 0.20, 0.50, and 0.80 were considered small, moderate, and large, respectively. A partial eta square of 0.01, 0.06, and 0.14 indicated a small, moderate, and strong effect [31]. A 2-way repeated measure analysis of variance with one in-between subject factor was calculated to determine differences between player’s dominance (dominant x non-dominant) across the two match formats (4v4 x 7v7) with Bonferroni’s *post-hoc* test. All statistical analysis were performed using SPSS for Windows (version 25, IBM Corporation, Chicago, IL, USA) and Microsoft Excel 2016 (Microsoft Corporation, Redmond, WA, USA).

## Results

The total number of actions was significantly higher in the 4v4 compared to the 7v7 match format (5.59 ± 1.44; 95%CI: 5.31–5.87 and 2.78 ± 0.73; 95%CI: 2.64–2.93, respectively, *d* = 2.76, *t*(102) = 21.38, *P* < 0.001). For all technical and tactical parameters, the 4v4 increased the number of ball-oriented actions compared to the 7v7 match format (Table 1). In particular, the 4v4 match format showed large effects (*d* = 0.84–2.48) in offensive parameters. Additionally, the 4v4 match format showed moderate to large effects (*d* = 0.40–1.87) for defensive parameters, i.e., successful duels and interceptions. A detailed analysis of the quality measures, i.e., ball control, pass, dribbling, shot and duel, showed a significantly increased number of successful (2.88 ± 0.92; 95%CI: 2.70–3.06 and 1.15 ± 0.49; 95%CI: 1.06–1.25, respectively, *d* = 2.34, *t*(102) = 21.48, *P* < 0.001) and unsuccessful actions (1.05 ± 0.42; 95%CI: 0.97–1.13 and 0.67 ± 0.23; 95%CI: 0.63–0.72, respectively, *d* = 1.14, *t*(102) = 8.76, *P* < 0.001) for the 4v4 compared to 7v7 match format.

**Table 1:** Quantity and quality of offensive and defensive technical-tactical parameters in the 4v4 and 7v7 match format per player per minute.

	4v4		7v7				
	n = 103		n = 103				
Parameters [per player per minute]	M ± SD	CI (95%)	M ± SD	CI (95%)	*t*-value	ES	Effect
Offensive							
**Ball control**	0.94 ± 0.43***	0.86; 1.03	0.38 ± 0.25	0.33; 4.23	*t*(102) = 13.10	1.62	large
Successful	0.86 ± 0.39***	0.78; 0.93	0.32 ± 0.23	0.27; 0.36	*t*(102) = 14.29	0.84	large
**Pass**	1.54 ± 0.50***	1.45; 1.64	0.67 ± 0.27	0.62; 0.73	*t*(102) = 17.32	2.17	large
Successful	1.20 ± 0.45***	1.11; 1.28	0.44 ± 0.21	0.39; 0.48	*t*(102) = 18.42	2.48	large
**Dribbling**	0.66 ± 0.46***	0.57; 0.75	0.34 ± 0.24	0.29; 0.39	*t*(102) = 8.57	0.88	large
Successful	0.46 ± 0.38***	0.38; 0.53	0.20 ± 0.16	0.17; 0.24	*t*(102) = 7.82	0.07	trivial
**Run with ball**	0.32 ± 0.21***	0.28; 0.36	0.11 ± 0.12	0.08; 0.13	*t*(102) = 10.09	1.24	large
**Shot**	0.30 ± 0.25***	0.25; 0.35	0.12 ± 0.11	0.10; 0.14	*t*(102) = 7.65	0.93	large
Successful	0.13 ± 0.13***	0.11; 0.16	0.03 ± 0.04	0.02; 0.03	*t*(102) = 8.70	0.49	moderate
Defensive							
**Duel**	0.49 ± 0.24***	0.44; 0.54	0.32 ± 0.15	0.29; 0.35	*t*(102) = 6.51	0.88	large
Successful	0.24 ± 0.16***	0.21; 0.27	0.17 ± 0.10	0.15; 0.19	*t*(102) = 4.18	1.87	large
**Interception**	0.33 ± 0.18***	0.30; 0.37	0.27 ± 0.15	0.24; 0.30	*t*(102) = 2.96	0.40	moderate
**Pressing**	1.01 ± 0.44***	0.92; 1.10	0.59 ± 0.24	0.54; 0.64	*t*(102) = 9.00	1.18	large

M: mean per player per minute; SD: standard deviation; CI: confidence interval; ES: effect size; parameters without qualitative (successful) evaluation were per se considered as a positive action for this age group, e.g. winning a ball through an interception was always evaluated positively. Significant differences between 4v4 and 7v7:

*, *P* < 0.05;

**, *P* < 0.01;

***, *P* < 0.001.

**Fig 1:**
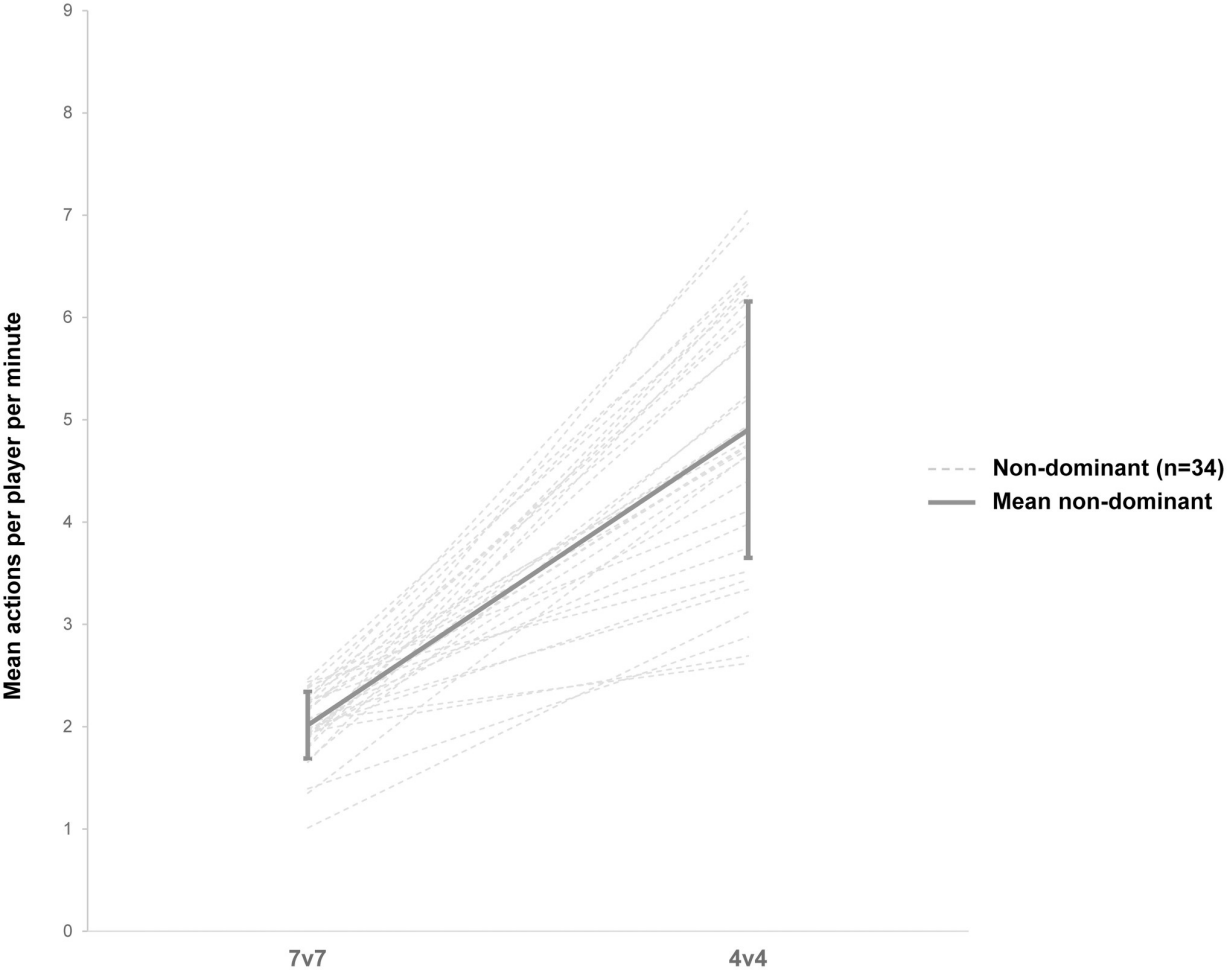
Number of actions per player per minute for dominant (n = 34) players in 7v7 compared to 4v4 match format.

**Fig 2:**
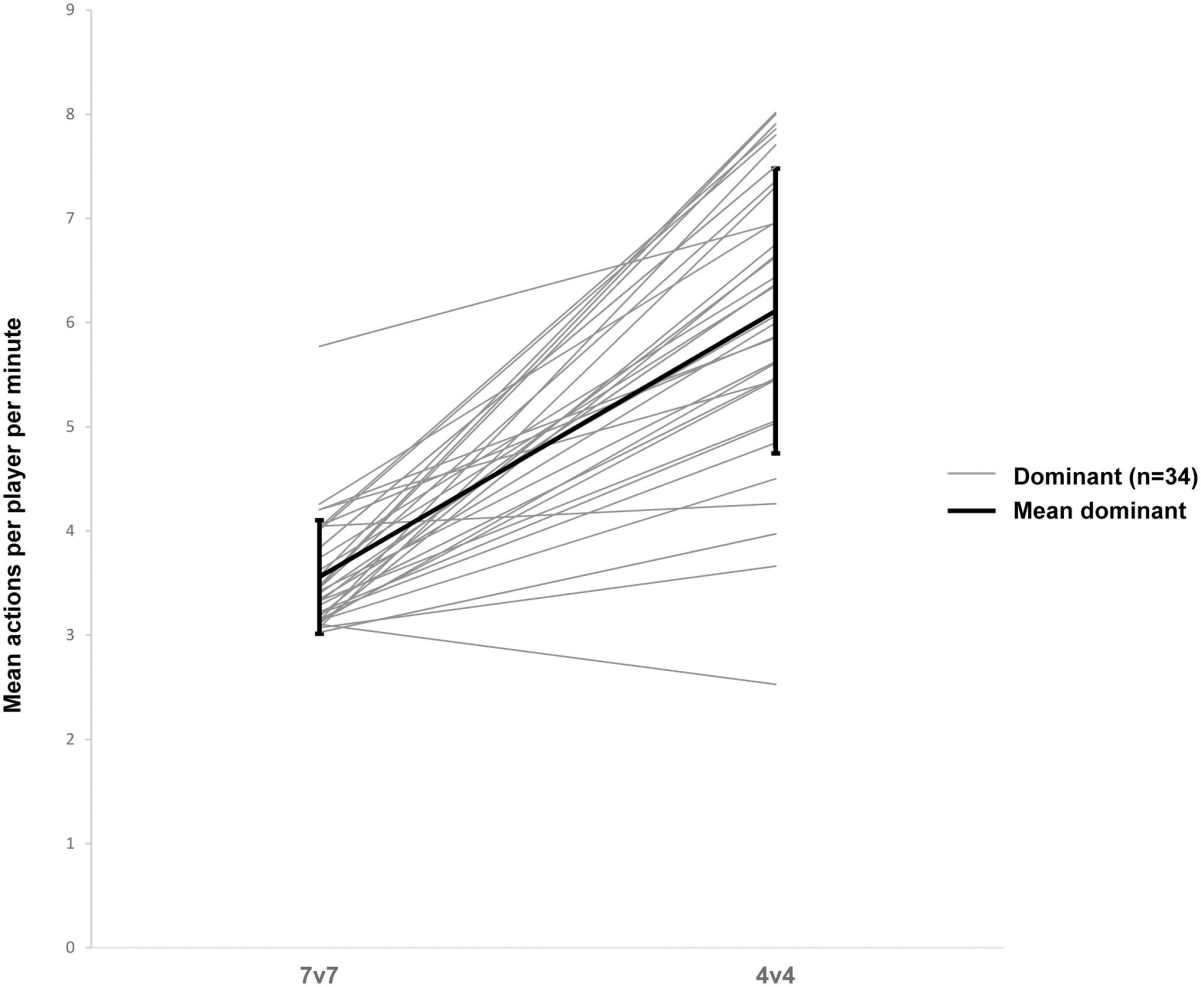
Number of actions per player per minute for non-dominant (n = 34) players in 7v7 compared to 4v4 match format.

Figs 1 and 2 illustrate total number of actions per player per minute during the 4v4 and 7v7 match formats for the dominant (Fig 1) and non-dominant (Fig 2) players. Compared to the 7v7, dominant players increased number of actions per player per minute by 72% from 3.56 ± 0.55 to 6.11 ± 1.37 (95%CI: 3.37–3.75 and 95%CI: 5.64–6.59, respectively, *d* = 2.46, *P* < 0.001) in the 4v4 match format. Non-dominant players increased their actions by 143% from 2.02 ± 0.33 to 4.90 ± 1.25 in the 4v4 (95%CI: 1.90–2.13 and 95%CI: 4.47–5.34, respectively, *d* = 3.16, *P* < 0.001). A detailed analysis of the technical and tactical actions of dominant and non-dominant players in 4v4 compared to 7v7 match format is presented in Table 2.

**Table 2:** The technical and tactical actions of dominant and non-dominant players in 4v4 match format compared to 7v7 (mean ± SD).

Parameters [per player per minute]		Dominant (n = 34)	Non-dominant (n = 34)		*F*-value	*P*-value	Partial eta^2^
Ball control	4v4	0.97 ± 0.54+	0.89 ± 0.37+	a)	*F*_(1,66)_ = 88	*P* < 0.001	0.57
	95%CI	0.85; 1.10	0.77; 1.02				
	7v7	0.56 ± 0.30*	0.25 ± 0.15	b)	*F*_(1,66)_ = 8	*P* = 0.006	0.11
	95%CI	0.44; 0.68	0.13; 0.37				
				c)	*F*_(1,66)_ = 4	*P* = 0.048	0.06
Pass	4v4	1.63 ± 0.48*+	1.39 ± 0.48+	a)	*F*_(1,66)_ = 208	*P* < 0.001	0.76
	95%CI	1.51; 1.76	1.26; 1.52				
	7v7	0.84 ± 0.26*	0.49 ± 0.19	b)	*F*_(1,66)_ = 18	*P* < 0.001	0.22
	95%CI	0.71; 0.97	0.37; 0.62				
				c)	*F*_(1,66)_ = 1	n.s.	
Dribbling	4v4	0.95 ± 0.53*+	0.46 ± 0.28+	a)	*F*_(1,66)_ = 63	*P* < 0.001	0.49
	95%CI	0.84; 1.06	0.35; 0.57				
	7v7	0.53 ± 0.22*	0.17 ± 0.09	b)	*F*_(1,66)_ = 45	*P* < 0.001	0.40
	95%CI	0.42; 0.64	0.06; 0.28				
				c)	*F*_(1,66)_ = 1	n.s.	
Run with ball	4v4	0.34 ± 0.21*+	0.25 ± 0.21+	a)	*F*_(1,66)_ = 54	*P* < 0.001	0.45
	95%CI	0.28; 0.40	0.20; 0.31				
	7v7	0.16 ± 0.16*	0.06 ± 0.07	b)	*F*_(1,66)_ = 9	*P* = 0.002	0.05
	95%CI	0.10; 0.22	0.00; 0.12				
				c)	*F*_(1,66)_ = 0	n.s.	
Shot	4v4	0.36 ± 0.27*+	0.26 ± 0.23+	a)	*F*_(1,66)_ = 41	*P* < 0.001	0.38
	95%CI	0.30; 0.42	0.19; 0.32				
	7v7	0.17 ± 0.12*	0.08 ± 0.09	b)	*F*_(1,66)_ = 8	*P* = 0.008	0.10
	95%CI	0.11; 0.23	0.01; 0.14				
				c)	*F*_(1,66)_ = 0	n.s.	
Duel	4v4	0.46 ± 0.24+	0.46 ± 0.25+	a)	*F*_(1,66)_ = 22	*P* < 0.001	0.25
	95%CI	0.39; 0.53	0.40; 0.53				
	7v7	0.34 ± 0.15	0.27 ± 0.13	b)	*F*_(1,66)_ = 1	n.s.	
	95%CI	0.27; 0.41	0.20; 0.33				
				c)	*F*_(1,66)_ = 1	n.s.	
Interception	4v4	0.35 ± 0.14*	0.27 ± 0.18+	a)	*F*_(1,66)_ = 5	*P* = 0.026	0.07
	95%CI	0.30; 0.40	0.22; 0.32				
	7v7	0.31 ± 0.16*	0.18 ± 0.12	b)	*F*_(1,66)_ = 18	*P* < 0.001	0.11
	95%CI	0.26; 0.36	0.13; 0.23				
				c)	*F*_(1,66)_ = 1	n.s.	
Pressing	4v4	1.05 ± 0.43+	0.92 ± 0.37+	a)	*F*_(1,66)_ = 56	*P* < 0.001	0.46
	95%CI	0.93; 1.16	0.81; 1.04				
	7v7	0.65 ± 0.29	0.52 ± 0.19	b)	*F*_(1,66)_ = 4	*P* = 0.041	0.06
	95%CI	0.54; 0.76	0.41; 0.63				
				c)	*F*_(1,66)_ = 0	n.s.	

Significant differences were identified with a 2-way ANOVA: dominance (dominant vs non-dominant) x match format (4v4 vs 7v7). a) Main effect: match format (4v4 vs 7v7); b) Main effect: dominance (dominant vs non-dominant); c) interaction effect: match format x dominance.

* Significant difference compared to non-dominant players;

^+^ Significant difference compared to 7v7;

n.s. not significant; CI confidence interval.

Quality assessment showed that dominant players completed more actions successfully than non-dominant players in both the 7v7 (65.1 ± 9.6%; 95%CI: 61.8–68.5% and 57.9 ± 11.3%; 95%CI: 54.0–61.9%, respectively, *d* = 1.00, *P* = 0.006) and 4v4 match format (74.8 ± 9.7%; 95%CI: 71.4–78.2% and 69.0 ± 9.6%; 95%CI: 65.7–72.3%, respectively, *d* = 1.06, *P* = 0.015). A detailed analysis revealed higher playing quality in dominant compared to non-dominant players during the 7v7 (*P* < 0.001), i.e., successful ball controls, successful passes, successful dribblings, and successful duels, as well as 4v4 matches (*P* < 0.001), i.e., successful passes and successful dribblings.

## Discussion

The main findings of the present study were that the 4v4 match format doubled the number of actions per minute per player compared to the traditional 7v7 match format in real game conditions. In particular, the offensive actions, i.e., ball control, pass, dribbling, run with ball and shot, were doubled, or even tripled when playing in a 4v4 match format. Additionally, the number of successful actions was significantly increased by the 4v4 compared to 7v7 match format (73% vs 62%). Dominant players showed significantly more actions of higher quality in both the 4v4 and 7v7 match format, compared to non-dominant players. However, the non-dominant players benefited from the 4v4 match format, as they showed a greater percentage increase in the mean number of technical and tactical actions compared to the dominant players (143% vs 72%, respectively).

With twice the number of technical and tactical actions per player, the 4v4 match format may double learning opportunities in children’s football. With the increased number of technical and tactical actions, the 4v4 is in line with the current literature that demonstrates significantly more technical and tactical actions on smaller pitches and with a reduced number of players [9]. In particular Garcia et al. (5v5, 7v7, 9v9) and Castelão et al. (3v3, 5v5) confirmed that reducing number of players in the field increased technical and tactical actions per player [11, 13]. Jones and Drust [14] showed a three-fold increase in the number of ball contacts in 4v4 compared to 8v8 training matches in elite youth football. They played the 4v4 match format on a 30 x 25 meter pitch with regular match play rules. Furthermore, fewer square meters per player in the 4v4 match format decreases space between players on the field which in turn increases number of duels, dribblings and interceptions [13, 32]. In the present study, the number of technical and tactical actions increased between 25% (interception) and 198% (run with ball) in the 4v4 compared to the traditional 7v7 match format.

Players could benefit from an increase in technical and tactical actions, as they involve quick decision-making in offensive and defensive match situations and, therefore, may improve skill acquisition. According to Serra-Olivares, Clemente and González-Víllora [1], decision-making should be developed in fast changing and flexible situations that mimic real match conditions. This perceptional and coordinative skill development is of particular interest in the early stages of a football player’s development [1, 33]. Therefore, younger players should be exposed to match formats that trigger these skills and the connections of these skills [34]. Ragarding the LET US Play principles of Brazendale et al. modifying rules in match formats to increase playing time of individual players could lead to higher physical activity in children compared to match formats using their traditional rules [35]. In addition to the improved learning opportunities, 4v4 match format allows for more dynamic resulting in a greater involvement of individual players, higher active game participation and, therefore, more commitment to and enjoyment of the game [36].

The number of successful actions in the 4v4, is significantly higher than in the 7v7 match format, with 73% compared to 62% respectively. Compared to the 7v7, there were more successful, but also unsuccessful actions per player per minute during the 4v4 match format. However, the reduced space between players and the less time between actions due to the smaller pitch may result in less efficient execution of actions [20].

As dominant players (best third; n = 34) executed 36% of all actions in the 4v4 and even 42% in the 7v7 match format, only 29% and 24% of all actions in the 4v4 and 7v7 match format respectively, are executed by the non-dominant players (the rest, 35% and 34%, was executed by the middle third). Nevertheless, in 4v4 with fewer players, non-dominant players had a greater increase (+ 71%) in the mean of players’ total amount of actions than the dominant players. According to Meylan et al. [21], all players should be involved in the game as equally as possible to allow equal chances of development for all players. As the percentage distribution was almost equal among all players in 4v4, especially non-dominant players may have increased game participation when match formats are adapted, leading to a more balanced game. This is highlighted by the data showing, that the player with the lowest number of actions (non-dominant group), reached 2.62 actions per minute in 4v4, while the player with the highest number of actions (non-dominant group) reached 2.47 actions per minute in 7v7 (Fig 2). Additionally, all players from the non-dominant group increased their number of actions in the 4v4 match format, suggesting a tendency to greater number of actions per minute in non-dominant players during the 4v4 match format. So far, the implementation of a 4v4 match format on game day in children’s football may balance the game participation among players.

When focusing on the quality of actions performed, the results showed that dominant players had significantly more successful actions in the 4v4 (*P* = 0.015) and in the 7v7 match format (*P* = 0.006) compared to non-dominant. More specifically, both passes and dribblings differed significantly between non-dominant and dominant players for both the 4v4 and the 7v7 match formats. However, successful shots did not differ between the two groups for the two match formats. Further, ball control and duels were both significantly higher for dominant compared to non-dominant players in the 7v7 match format but did not differ in the 4v4. Non-dominant players so far benefited from more successful ball controls and duels in the 4v4 compared to the 7v7 match format. Despite the fact that non-dominant players may benefit from the 4v4 match format, the present study is the first to analyze game involvement regarding players’ dominance. However, more research is needed to analyze players’ game involvement based on position specificity.

A limitation of the study is that various task constraints (pitch size, number and size of targets and number of players) were changed. Therefore, effects cannot be subdivided and different influences of the constraints on the results cannot be estimated. Additionally, due to different playing time in 4v4 and 7v7, a possible fatigue effect towards the end of a game cannot be completely excluded [37]. Furthermore, only the acute effect of the 4v4 match format was investigated. As such, future studies should quantify the effect of the 4v4 match format during a prolonged training and competition period and evaluate players’ long-term development.

## Conclusion

The 4v4 match format doubled the number of actions per player per minute compared to the traditional 7v7 match format. In addition, the number of successfully actions was significantly increased in the 4v4 match format (73% vs 62%), and both dominant and non-dominant players executed significantly more actions. Despite a missing significant interaction effect, there was a larger percentage increase in number of actions for the non-dominant players (143%) compared to dominant players (72%) in 4v4. This match format improves involvement of each player and results in higher active game participation under real game conditions. As such, learning opportunities for children football players are increased, which may benefit skill and talent development.

## Supporting information

S1 Fig Player’s value distribution of the mean number of actions per minute in 7v7. (TIF)

S1 File Raw data Table 2. (XLSX)
