# Supplementary figures and images for: How to improve technical and tactical actions of dominant and non-dominant players in children’s football?
Source: PLoS One. 2021 Jul 22;16(7):e0254900. doi: 10.1371/journal.pone.0254900 (PMC8297913; doi:10.1371/journal.pone.0254900)

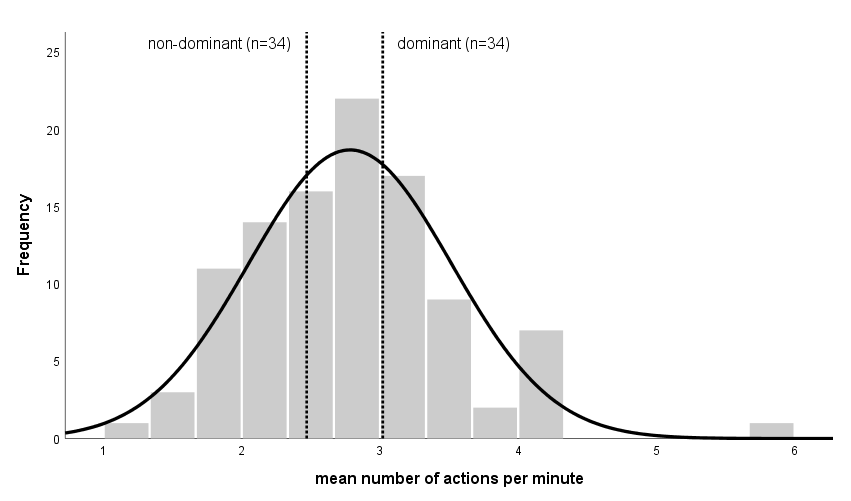

Supplement: S1 Fig — (TIF) [file pone.0254900.s001.tif]
